# Endoparasites of Stray Dogs in Mashhad, Khorasan Razavi Province, Northeast Iran with Special Reference to Zoonotic Parasites

**Published:** 2013

**Authors:** Amir ADINEZADEH, Eshrat Beigom KIA, Mehdi MOHEBALI, Saideh SHOJAEE, Mohammad Bagher ROKNI, Zabihollah ZAREI, Golamreza MOWLAVI

**Affiliations:** 1 Department of Medical Parasitology and Mycology, School of Public Health, Tehran University of Medical Sciences, Tehran, Iran; 2 Center for Research of Endemic Parasites of Iran (CREPI), Tehran University of Medical Sciences, Tehran, Iran; 3 Meshkin-Shahr Health Station, National Institute of Health Research, Iran

**Keywords:** Helminth, Tissue Protozoa, Stray Dog, Iran

## Abstract

**Background:**

To find out different species of helminthes and blood/tissue protozoan parasites of stray dogs and their potential role for transmission of zoonotic species to human in Mashhad, Khorasan Razavi Province, northeast Iran, during 2008-2009.

**Methods:**

Totally, 100 stray dogs were selected among Mashhad municipal collection from different sites of the city. Internal organs were examined for any parasites. Helminthes were identified based on morphological characteristics. Smears prepared from peripheral blood as well as liver, spleen and any skin lesion were stained by Giemsa and examined microscopically. Samples obtained from spleen were aseptically cultured in three culture media including NNN, Schneider's Drosophila (HIMEDIA) and RPMI1640 (GIBCO) for isolation of *Leishmania* spp. The titer of anti-*Leishmania* and anti-*Toxoplasma* antibodies were measured by direct agglutination test (DAT) and indirect fluorescent antibody test (IFAT), respectively.

**Results:**

84% of dogs were infected at least with one species of intestinal helminthes. The species of parasites and rate of infection were as follows: *Taenia hydatigena* (61%), *Dipylidium caninum* (46%), *Mesocestoides lineatus* (19%), *Echinococcus granulosus* (10%), *Toxascaris leonina* (53%) and *Toxocara canis* (7%). Anti-*Leishmania* antibodies were detected by DAT in 8 dogs (8%) at 1:320 titers and higher. Forty seven dogs (47%) showed anti-*Toxoplasma* titer at 1:10 and 17 (17%) showed titer of ≥1:100. No blood parasites were found in prepared blood smears.

**Conclusion:**

The high rate of parasitic infection and presence of zoonotic species especially *E. granulosus* and *T. canis* emphasizes the risk of diseases spread in urban areas by stray dogs

## Introduction

There are a wide variety of parasites infecting dogs, among those several parasites have the potential of infection for humans. *Echinococcus granulosus*, etiological agent of cystic echinococcosis (CE) or hydatid disease in humans and livestock, is the most important zoonotic helminthes. Adult worms live in intestine of dogs, producing eggs which pass with feces into the environment. The rates of CE vary in different geographical regions, but are linked to the prevalence of adult worm infection in domestic, stray and wild dogs (1). Review on previous studies in Iran indicate the occurrence of *E. granulosus* with different prevalences in several definitive hosts (2–7) and intermediate animal hosts (2, 5–7), as well as human hydatidosis (5, 6) in different parts of the country.

*Toxocara canis* and *Toxascaris leonina* are common ascaridoid nematodes of dogs throughout the world. *T. canis* has important role in human toxocariasis. Humans are infected by the ingestion of eggs. There is a clear connection between the degree of soil contamination with *Toxocara* eggs and the prevalence of toxocariasis among people (8). *T. canis* in dogs has been reported with different prevalences in Iran (3, 4, 7, 9), and seropositivity in human especially children is also recorded (9).

Dogs are also source of several zoonotic protozoan parasites especially *Leishmania infantum* which infects human via females sandfly bite. Domestic dogs are the principal animal reservoir hosts for *L. infantum* in both old and new worlds (10). In urban areas that stray dogs roam freely around human settlements, there is the risk of spreading of infectious agents in the environment and transmission of these zoonotic diseases to human.

Mashhad metropolitan city in Khorasan Razavi Province, northeast Iran attract millions of visitors from different parts of the country, as well as other countries all throughout the year. Infectivity of stray dogs in this city can be health treat both for residents and travelers. Previous study in this city indicated high prevalence of intestinal helminthes in stray dogs (3). However, the population of stray dogs is still high and no study has been documented on examination of this reservoir host considering probable occurrences of extra intestinal helminthes and protozoan parasites. Therefore, Mashhad municipality campaign for control of stray dogs’ population provided an opportunity for such study.

Accordingly, the aim of the present study was to find out the variation of helminth and zoonotic protozoan tissue parasites in stray dogs in this city in relation with different criteria with special attention to zoonotic species.

## Materials and Methods

### Study area

Mashhad (36° 18′ N, 59° 36′ E), the center of Khorasan Razavi Province and the second largest city of Iran, is situated in northeastern part of the country, near the borderlines of Afghanistan and Turkmenistan. The total area is 118854 km^2^, with steppe climate, having cool winters, pleasant springs, mild summers and beautiful autumn. It is the holiest city in the country with a population of about 2.5 million and 20 million visitors every year (11).

### Sampling

During a program performing by Mashhad municipal on slow killing of stray dogs, using euthanizing drugs, after communication and correspondence with Khorasan Razavi Health Centre 100 dogs were selected for this cross sectional study. Collection was carried out by simple random sampling in warm season (from April to September) and cold season (from October to March) from different cites of the city, during 2008-2009. In field laboratory for each dog, different characters including sex, color of fur, and season of captivity were recorded. Based on the life stage, dogs were grouped by the veterinarian as puppy (less than two years old) or adult (more than two years old). Also, clinical signs including hair lose, lethargy, emaciation or presence of scar was also registered, in case of observation. Thick and thin blood smears were prepared, in addition to separation of sera and storage at -20 °C. Necropsy was performed soon after death. Impression smears of liver, spleen and skin lesion were prepared rapidly, as well as aseptically collection of liver and spleen specimens for the purpose of culture. Different internal organs were macroscopically screened for parasites. All the samples were preserved and transported to the School of Public Health, Tehran University of Medical Sciences for further parasitological examinations, using specific techniques.

The study was approved by Ethics Committee of Tehran University of Medical Sciences.

### Examination methods

Blood thick and thin smears as well as impression smears of liver, spleen and skin lesion were stained by Giemsa and examined microscopically with high magnification (1000X). Biopsy specimens, collected aseptically from the liver and spleen of the dogs were cultured in three culture media including NNN, Schneider's Drosophila (HIMEDIA) and RPMI1640 (GIBCO), containing 10% fetal calf serum and 100-200 UI/ml penicillin G and 1 ug/ml streptomycin, for isolation of *Leishmania* spp. The cultures were incubated at 26 °C for up to six weeks and examined weekly for the demonstration of promastigotes of *Leishmania*. Sera were examined by direct agglutination test (DAT) for *Leishmania* infection as described by Mohebali et al. (12, 13) and by indirect fluorescent antibody (IFA) test for *Toxoplasma* as reported by Voller & O'Neill (14).

Thin slices of diaphragm and skeletal muscles were prepared manually and after staining with acetic carmine examined microscopically for infectivity with *Sarcocystis* or *Trichinella*. Small and large intestines were dissected separately and after macroscopical observation, small intestines were examined using intestinal scraping technique for *E. granulosus* as described by Deplazes and Eckert (15).

Contents of stomach, small and large intestines were washed through sieves, under pressure of tap water, and sediments were preserved in 10% formalin. After clearing and specific staining of helminth specimens (7), identification of these parasites carried out based on morphological characteristics using valid references for nematodes (7) and cestodes (16).

For statistical analysis Chi-square and Fisher exact tests were applied. A *P*-value of less than 0.05% was considered as significant probability.

## Results

Stray dogs (n = 100) collected by Mashhad municipality in cold season (n= 60) and warm season (n= 40) were examined for helminthic parasites and tissue-blood protozoan parasites in relation with different criteria including sex, life stage, and fur color.

Considering the infectivity of the dogs, in general 87% were infected with at least one helminth or protozoan parasite. Table 1 demonstrates the frequency and prevalence of infectivity with helminthic parasites. Accordingly, 84% (95% confidence interval (CI) = 76.81-91.19) of the dogs were infected with at least one helminth parasite.

**Table 1:** Frequency and prevalence of infectivity with different helminth parasites in Mashhad stray dogs according to different criteria

Criteria	Season n (%)		Sex n (%)		Fur color n (%)	
	Warm (n = 40)	Cold (n = 60)	Male (n = 63)	Female (n = 37)	Light (n = 74)	Dark (n = 26)
***Taenia*** ***hydatigena***	27 (67.5)	34 (57)	39 (62)	22 (59)	45 (61)	16 (61)
***Dipylidium*** ***caninum***	20 (50)	26 (43)	33 (52)	13 (35)	35 (47)	11 (42)
***Mesocestoides*** ***lineatus***	6 (15)	13 (21.7)	12 (19)	7 (19)	17 (23)	2 (7.7)
***Echinococcus*** ***granulosus***	3 (7.5)	7 (11.7)	4 (6.3)	6 (16.2)	7 (9.5)	3 (11.5)
***Toxascaris*** ***leonina***	22 (55)	31 (52)	35 (55.5)	18 (48.6)	44 (59)	9 (34.6)
***Toxocara*** ***canis***	5 (12.5)	2 (3.3)	4 (6.3)	3 (8.1)	5 (6.7)	2 (7.7)

The most and the least prevalent helminth was *Taenia hydatigena* (61%) (95% CI = 51.4-70.6) and *T. canis* (7%) (95% CI = 2-12), respectively. Statistical analysis indicated no significant difference (*P* ≥ 0.05) between infectivity of any helminth recovered and different criteria including season, sex, and fur color. However, P-values for rate of infection with *Mesocestoides lineatus* and *To. leonina* in relation with fur color were 0.07% and 0.05%, respectively, both with higher infectivity in dogs with light fur color. Since only a few dogs were puppy (n = 4) life stage criteria was not included in Table 1.

The results of DAT related anti-*Leishmania* sero-positive dogs in association with different criteria are illustrated in Table 2. As this table indicates, 8% of the stray dogs (95% CI = 0.68-15.32) had anti-*Leishmania* antibodies with the titer of 1:320 and over. Moreover, none of the dogs with clinical signs was found seropositive; on the other hand, all 8 seropositive dogs were asymptomatic and most of them (n = 7) related to cold season. Statistical analysis revealed no significant differences between seropositivity of the stray dogs and any different criteria including season (*P* = 0.1), sex (*P* = 0.62), life stage (*P* = 0.71), fur color (*P* =0.34) and clinical signs (*P* = 0.59). However, there was significant difference between clinical signs and season (*P* = 0.01), i.e. most of the dogs with clinical signs were related to warm season, while seropositivity was significantly higher in cold season (*P* = 0.001).

**Table 2:** Sero-prevalence of the stray dogs with anti-*Leishmania* antibodies by direct agglutination test (DAT) in association with different criteria

	Clinical signs** n (%)		Gender n (%)		Life stage n (%)		Fur color n (%)		Season n (%)	
	Positive	Negative	Male	Female	Adult	Puppy	Light	Dark	Warm	Cold
**DAT** ^+^	0 (0)	8 (9.1)	5 (7.9)	3 (8.1)	8 (8.3)	0 (0)	5 (6.6)	3 (11.5)	1 (2.5)	7 (11.7)
**DAT** ^–^	12 (100)	80 (90.9)	58 (92.1)	34 (91.9)	88 (91.7)	4 (100)	69 (93.4)	23 (88.5)	39 (97.5)	53 (88.3)
**Total**	12	88	63	37	96	4	74	26	40	60

* Positive titer ≥ 1:320/

** Clinical signs included on hair lose, lethargy, emaciation and presence of scare

The results of anti-*Toxoplasma* antibodies in the dogs by IFA test indicated that 47%, 6% and 11% of the dogs had anti-*Toxoplasma* antibodies at the titer of 1:10, 1:100 and 1:200 or over, respectively. Accordingly, geometric mean of reciprocal of antibody titre (GMRT) was calculated as 1: 20.

No helminthes or protozoan parasite was detected in diaphragm or skeletal muscles.

## Discussion

In this study, among 100 stray dogs, 87% had infectivity with at least one helminthes or protozoan parasite. Considering the helminth parasites 84% were infected. For none of the helminth species there was significant difference between male and female dogs, and this is correspondent with the result of the previous studies (3, 4, 17). The most prevalent helminth parasite in the current study was *T. hydatigena* (61%). Infectivity of dogs with this helminth, with higher prevalence in older dogs, has also been reported by other researchers (3, 4). The higher occurrence of this helminth in older dogs is most probably as a result of longer exposure of older dogs with the source of infection and longevity of the worms. In contrast, *T. canis* was the least prevalent helminth in this study (7%); while in a previous study on Mashhad dogs the rate of infection with *T. canis* was higher (39%) (3). This difference is due to the age effect; because in the current study most animals were adult, and in such dogs *T. canis* larvae undergo a somatic migration to the tissues (18), while in puppies they settle in the intestine and mature to adults.

In the present study, 10% (95% CI = 4.1-15.9) of the stray dogs were found infected with *E. granulosus*. This parasite is prevalent in stray dogs in different parts of the country as 5% to 49% (6). In addition, it has been reported from other animals including domesticated dogs, golden jackals, wolves, and red fox with different prevalences and intensity of infection (5). Human cases are also regularly reported with the rate of 0.6-1.2/100000 (5, 6), as well as infectivity of different intermediate hosts (5–7).

Considering the protozoan tissue parasites, infectivity of the stray dogs with *Leishmania* and *T. gondii* infections were detected in the present study. Detection of canine *L. infantum* infection is necessary to define control measures for zoonotic visceral leishmaniasis (VL) (10). According to previous studies (12, 19–21), DAT has been introduced as an appropriate serological test for detection of *L. infantum* infection in humans and dogs. Therefore, in the current study, DAT was applied for determination of sero-prevalence of canine *L. infantum* infection. From 100 serum samples collected from stray dogs, 8 cases were sero-positive by DAT in titers of ≥1:320; but none of the sero-positive dogs showed any signs. Majority of dogs can remain infected by *L. infantum* without displaying apparent clinical signs of VL for years even for their entire life (22). It is estimated that more than 50% of the seropositive dogs identified in field surveys are asymptomatic (12, 23). The role of asymptomatic dogs, as a source of infection to human and susceptible animals, is still unknown. Some investigators stated that asymptomatic dogs have no important role in the transmission of leishmaniasis; because they had little or no capacity to infect vectors (24). But the results reported by Molina et al.^25^, Alvar et al. (26) and Moshfea et al. (27) showed the epidemiological importance of the subclinical period of the infected animals, during the time that they did not show any clinical signs. Therefore, sero-positive dogs in this study are capable of infecting the vectors and are essential risk factors for human infection in the area.

Respect to the fur size criteria, higher percent of seropositivity against anti-*Leishmania* antibodies were found in dogs with dark fur (11.5%) than that of light fur (6.6%); however, this difference was not statistically significant. Discrimination between different lights by *Lutzomia longipalpis”* vector of *Leishmania* parasite in southern areas of the Nearctic” has been tested; indicating that these sand flies can discriminate lights with different intensity and wavelength and thus have true color vision (28).

Among 100 tested sera, 36 were negative for *T. gondii* antibody; among seropositive dogs, 17 dogs had anti *T. gondii* antibody titers of 1/100 and over. *T. gondii* antibodies have been found in canine sera worldwide. Clinical signs of *T. gondii* infection in dogs are variable and include respiratory, digestive, ocular, neurological and muscular disturbances (29). Seroprevalence increases with age and is higher in older dogs as well as dogs from rural areas (30). IgM antibodies were found in 2% of 658 dogs, and in a logistic regression model it was shown that older or heavier dogs had higher odds of seropositivity than younger or lighter ones and mixed bred dogs had higher odds of seropositivity than pure bred dogs (31). In a study on German shepherd dogs in Korea, *T. gondii* DNA in sera of 64 out of 138 dogs (46.3%) were detected by nested PCR assay with no differences in gender and age (32). In Iran, few studies have been performed to evaluate *T. gondii* infection in animals; for example, Ghorbani et al. (33) performed latex agglutination test and found the highest seropositive rate of *T. gondii* infection in stray dogs among different animals tested including 111 cats, 113 dogs, 3 jackals, 393 sheep, 272 goats and 69 cows. The seropositive rate ranged from 12.6 to 56.0% in these animals; the highest (56.0%) was in the stray dogs. In another study in west and central parts of Iran, from a total of 548 dogs, 159 (29%) were positive for *Neospora caninum* and 147 (26.8%) for *T. gondii*. No sex predisposition was detected in these dogs, but age and living places had important role for both *N. caninum* and *T. gondii* infections (34). In Tehran, it was shown that 22.47% of 89 tested dogs had antibodies against *T. gondii* infection, with significantly higher rate in dogs older than one year old, without any sex predisposition (35). Therefore, it seems that the relatively high seroprevalence of *T. gondii* infection in dogs in Iran can be due to high population of stray dogs and environmental contamination.

## Conclusion

As Mashhad is a metropolitan city, attracting millions visitors all throughout the year, infectivity of stray dogs with different helminth and protozoan parasites, especially *E. granulosus*, *T. canis*, and *L. infantum* is a significant public health concern. Future epidemiological studies will clarify the role of reservoir host fur color as a risk factor for attracting of phlebotomine sand flies and therefore increasing transmission of canine leishmaniasis. Any control program on mentioned zoonotic parasites in this tourism scenic focus of the country by municipality and health authorities requires considering different environmental measures and management of the diseases in human and animals hosts especially stray dogs, as well as control of vectors in order to prevent the transmission of infectious agents to human and animal hosts.
